# Cardiac tamponade related to a coronary injury by a pericardial calcification: an unusual complication

**DOI:** 10.1186/1471-2261-12-28

**Published:** 2012-04-25

**Authors:** Anne Cypierre, Francis Pesteil, Claude Cassat, François Parraf, Rémy Bellier, Lionel Ursulet, Claire Eveno, Philippe Vignon, Bruno François

**Affiliations:** 1 Service de Réanimation Polyvalente, CHU Dupuytren, 2 avenue Martin Luther King, Limoges 87042, France; 2 Service de Chirurgie Thoracique et Cardio-vasculaire, CHU Dupuytren, 2 avenue Martin Luther King, Limoges 87042, France; 3 Service de Cardiologie, CHU Dupuytren, 2 avenue Martin Luther King, Limoges 87042, France; 4 Service d'Anatomopathologie, CHU Dupuytren, 2 avenue Martin Luther King, Limoges 87042, France

**Keywords:** Hémopéricardium, Tamponade, Chronic péricarditis, Coronary artery

## Abstract

**Background:**

Cardiac tamponade is a rare but severe complication of pericardial effusion with a poor prognosis. Prompt diagnosis using transthoracic echocardiography allows guiding initial therapeutic management. Although etiologies are numerous, cardiac tamponade is more often due to a hemopericardium. Rarely, a coronary injury may result in such a hemopericardium with cardiac tamponade. Coronary artery aneurysm are the main etiologies but blunt, open chest trauma or complication of endovascular procedures have also been described.

**Case presentation:**

A 83-year-old hypertensive man presented for dizziness and hypotension. The patient had oliguria and mottled skin. Transthoracic echocardiography disclosed a circumferential pericardial effusion with a compressed right atrium, confirmed by contrast-enhanced thoracic CT scan. A pig-tail catheter allowed to withdraw 500 mL of blood, resulting in a transient improvement of hemodynamics. Rapidly, recurrent hypotension prompted a reoperation. An active bleeding was identified at the level of the retroventricular coronary artery. The pericardium was thickened with several "sharping" calcified plaques in the vicinity of the bleeding areas. On day 2, vasopressors were stopped and the patient was successfully extubated. Final diagnosis was a spontaneous cardiac tamponade secondary to a coronary artery injury attributed to a "sharping"calcified pericardial plaque.

**Conclusion:**

Cardiac tamponade secondary to the development of a hemopericardium may develop as the result of a myocardial and coronary artery injury induced by a calcified pericardial plaque.

## Background

Cardiac tamponade is a life-threatening complication of pericardial effusions. Prompt diagnosis using transthoracic echocardiography allows guiding initial therapeutic management [1,2]. The etiology of cardiac tamponade reflects various conditions that cause pericardial effusions, trauma or the rupture of the heart [3]. We herein report on a patient presenting with cardiac tamponade secondary to a myocardial and coronary artery injury related to an erosive pericardial calcification, who had a favorable outcome after surgical decompression.

## Case presentation

**Figure 1:**
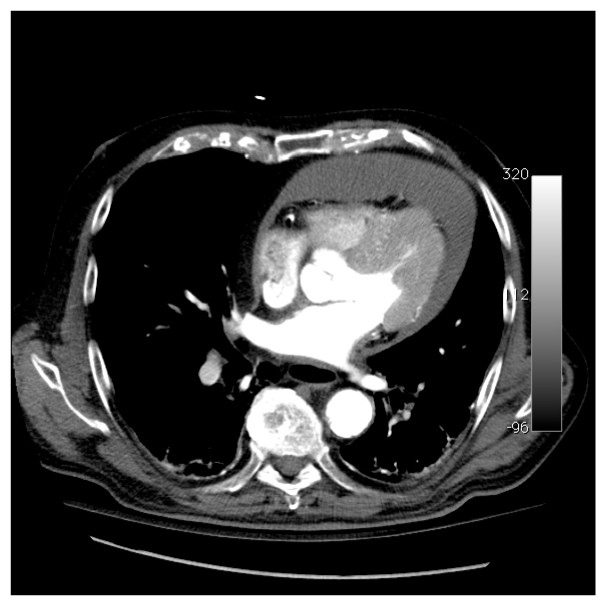
**Contrast-enhanced thoracic CT scan: circumferential pericardial effusion without other anormalities, in particular aortic lesion**.

**Figure 2:**
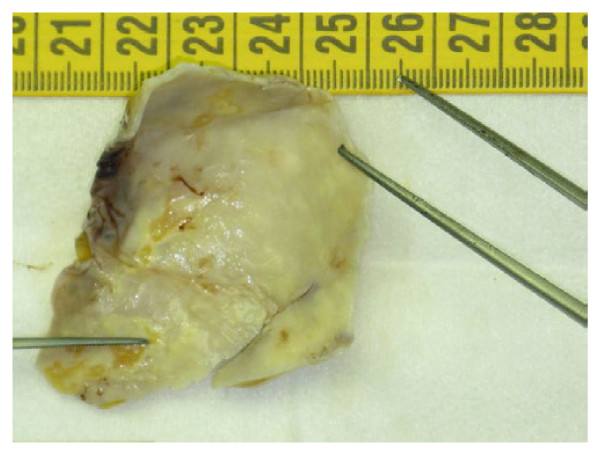
**Piece of pericardiectomy: calcified pericardial plaque of parietal posterior pericardium**.

A 83-year-old hypertensive man presented to the Emergency Department for dizziness and hypotension. He was treated by β-blockers (bisoprolol), diuretics (hydrochlorothiazide), ACE inhibitors (valsartan) and platelet inhibitors (lysine acetylsalicylate) for hypertension and arythmia. The patient denied any thoracic pain or recent trauma. Upon admission, blood pressure was 60/40 mmHg on both arms, and hypotension persisted despite a fluid loading of 2.5 L. A vasopressor support was promptly initiated (norepinephrin: 1,2 μg/kg/min). A bradycardia (54 bpm) with decreased cardiac sounds and distended jugular veins were noted. The patient had oliguria and mottled skin. A severe metabolic acidosis was observed (pH: 7.31; BD: -10.4 mmol/L; lactate: 6.76 mmol/L). ALAT level was moderately increased (62 UI/L) without increase in bilirubin or troponin. The electrocardiogram recorded a normal sinus rhythm with an incomplete left bundle branch block. Transthoracic echocardiography disclosed a circumferential pericardial effusion with a compressed right atrium and increased respiratory variations of tricuspidal mitral Doppler velocities. Left ventricular systolic function was normal, without regional wall motion abnormality. Contrast-enhanced thoracic CT scan ruled out an acute dissection of the ascending aorta and confirmed the presence of the circumferential pericardial effusion (Figure 1). A pig-tail catheter was placed within the pericardial sac using the subcostal approach under echocardiographic guidance. There were withdrawn 500 ml of blood, which resulted in a transient improvement of hemodynamics. Rapidly, hypotension resumed despite increasing doses of Norepinephrine (up to 0,7 μg/kg/min) and the pericardial drainage remained productive (450 ml/hour of fresh blood). This prompted a reoperation under extracorporeal circulation. The surgeon confirmed the presence of a hemopericardium with numerous clots in the dependent region of the pericardial sac. An active bleeding was identified at the level of the retroventricular coronary artery and of the epicardial surface which was related to a superficial laceration of the posterolateral wall of the left ventricle. The pericardium was thickened with several "sharping" calcified plaques in the vicinity of the bleeding areas. Hemostatic patches were placed and the posterior aspect of the pericardium was resected and replaced by a pericardial patch. The postoperative course was uneventful. On day 2, vasopressors were stopped and the patient was successfully extubated. The pathologic examination of pericardial plaques disclosed a calcified pericardium without specific tumoral infiltration or inflammatory process (Figure 2). No any sign of a tuberculosis origin was evidenced. One month later, the patient remained asymptomatic. Final diagnosis was a spontaneous cardiac tamponade secondary to a coronary artery injury attributed to a "sharping"calcified pericardial plaque.

## Discussion

Tamponade results in a circulatory failure secondary to the compression of cardiac cavity by a pericardial effusion increasing the pericardial pressure. Transthoracic echocardiography is key in confidently diagnosing cardiac tamponade. Main echocardiographic findings, as reported in the present case, include the diastolic compression of right cardiac cavities, the dilatation of the inferior vena cava without respiratory variations of its diameter, and increased respirator variations of intracardiac Doppler velocities [1,4-6]. In addition, echocardiography may also identify the origin of cardiac tamponade and help guiding the pericardocentesis, as in our patient.

Lethal cardiac tamponade is frequently related to a hemopericardium which may be related to a ruptured abnormal ascending aorta (e.g., acute aortic dissection) or to a complicated acute myocardial infarction (i.e., wall rupture) [3,7]. In a necropsy series including 461 patients who died from cardiac tamponade, the volume of hemopericardium varied between 150 and 1000 mL [3]. Accordingly, the identification of a hemopericardium is a warning sign preceding a lethal cardiac or vascular wall rupture or rapidly progressing tamponade.

In patients who reach the hospital alive, cardiac tamponade secondary to a hemopericardium is most frequently related to therapeutic invasive procedures (31% of the cases), whereas other etiologies are related to cancer (26%), acute myocardial infarction (11%) or has been reported as essentials (10%) [8].

Rarely, a coronary injury may result in a hemopericardium and cardiac tamponade. In those cases, reported etiologies are coronary artery aneurysm [9] with an incidence of 0.3 to 4.2% which may be related to atherosclerosis in 50 to 90% of the cases [7], chest trauma [10], localized infections [11], or may develop spontaneously [12]. Injury to coronary arteries leading to a hemopericardium have also been described after blunt or open chest trauma, or as a complication of endovascular procedures such as coronary angiography [13]. As far as we know, we report herein the first case of hemopericardium secondary to a myocardial and coronary artery injury induced by a calcified pericardial plaque.

## Conclusion

Cardiac tamponade secondary to the development of a hemopericardium may develop as the result of a myocardial and coronary artery injury induced by a calcified pericardial plaque.

## Consent

Written informed consent was obtained from the patient for publication of this case report and accompanying images.

## Competing interests

The authors declare that they have no competing interests.

## Authors' contributions

AC was a member of the ICU team and wrote the paper. FPe performed the surgery and helped writing the paper. CC performed the echography and the pericardial drainage. FPa was the anathomopathologist. CE was a member of the surgical team. RB, LU, PV and BF were members of the ICU team. PV and BF helped in manuscript writing, and performed the final control of the paper. All authors have read and approved the final manuscript.

## Pre-publication history

The pre-publication history for this paper can be accessed here:

http://www.biomedcentral.com/1471-2261/12/28/prepub
